# Technological Evaluation of Fiber Effects in Wheat-Based Dough and Bread

**DOI:** 10.3390/foods13162582

**Published:** 2024-08-18

**Authors:** Celeste Verbeke, Els Debonne, Stien Versele, Filip Van Bockstaele, Mia Eeckhout

**Affiliations:** 1 Safety and Health, Research Unit Cereal and Feed Technology, Department of Food Technology, Faculty of Bioscience Engineering, Ghent University, Valentin Vaerwyckweg 1, 9000 Ghent, Belgium; celeste.verbeke@ugent.be (C.V.);; 2 Safety and Health, Food Structure and Function Research Group, Department of Food Technology, Faculty of Bioscience Engineering, Ghent University, Coupure Links 653, 9000 Ghent, Belgium; filip.vanbockstaele@ugent.be

**Keywords:** wheat dough, wheat bread, fiber substitution, dough rheology, bread quality, texture, PCA, functionality

## Abstract

Dietary fiber incorporation in bread offers potential health benefits but poses challenges due to its impact on dough rheology and bread quality. This study evaluated the effects of pea, cocoa, and apple fiber on wheat-based dough and bread properties using rheological methods (farinograph, alveograph, pasting, and proofing) and baking trials. Substituting flour with fiber at 1%, 5%, or 10% increased water absorption and affected dough development, stability, and extensibility, particularly at high fiber concentrations. Pasting properties showed varying gelatinization behaviors influenced by fiber type and concentration. Principal component analysis (PCA) highlighted the clustering of dough and bread characteristics based on fiber concentration and type. At low fiber concentrations (up to 5% of flour replacement), negative effects were minimal, suggesting no need for comprehensive compositional analysis. However, high fiber concentrations (10%) introduced significant variability and complexity in dough properties. New farinographic parameters (FU_4_, FU_6_, FU_8_, FU_10_, and FU_12_) improved the explanatory power of PCA, enhancing the understanding of fiber-rich dough dynamics. The significant alterations in moisture content and texture underscore the intricate relationship between type of fiber, concentration, and dough functionality. Optimizing rheological parameters for fiber-enriched flour is crucial for adapting the bread-making process to produce high-quality bread with desired characteristics and enhanced nutritional benefits.

## 1. Introduction

Numerous scientific studies have explored the enrichment of bread products with dietary fiber, underscoring the potential nutritional benefits of such enhancements [1,2,3,4,5,6,7]. Fiber enrichment of bread is highly promising due to the well-documented positive correlation between fiber consumption and the reduction in coronary heart disease and diabetes incidence [8,9]. A study by Wu et al. [9] showed that increased consumption of soluble and insoluble fibers has a similar effect on lowering the risk of coronary heart disease, especially for fibers derived from cereals and fruits. Currently, the fiber-enriched breads available in retail stores are predominantly based on cereal dietary fibers, such as fiber-rich wholegrain products made from wheat, spelt, rice, oats, barley, and rye [10,11]. These sources are widely utilized due to their availability and compatibility with traditional bread-making processes [12,13]. Further, there is a notable underrepresentation of fibers derived from fruits and vegetables in commercially available bread products, despite their presence in the scientific literature [4,5,6,14,15]. Another group of ingredients more frequently used in bread applications are legumes (e.g., bean, chickpea, lentil, lupin, and pea). This group is nutritionally recognized for its high fiber and protein content [16]. In practice, legumes are mostly added to bread formulations due to their techno-functional properties, rather than their nutritional benefits [17,18].

The main challenge in fiber enrichment of bread lies in the intrinsic properties of dietary fibers, particularly their water-holding capacity [19,20]. This characteristic can lead to a competitive interaction between the added fibers and the wheat gluten for water, impeding the gluten functionality and thus often resulting in a denser and firmer bread product [20]. However, increased water retention can be beneficial in the context of anti-staling, bake-off technology, and gluten-free bread production [21,22,23,24]. Hydrocolloids such as alginate, carrageenan, hydroxypropyl methylcellulose (HPMC), carboxymethyl cellulose (CMC), gum arabic, locust bean gum, xanthan, guar gum, and pectin are polysaccharides that act as gelators or thickeners when dispersed in water. Their ability to form stable gels helps to maintain bread structure and prevent moisture redistribution during storage [24,25]. Additionally, the properties of hydrocolloid substances are particularly valuable in frozen dough and par-baking technology with frozen storage in retaining moisture during freeze–thaw cycles, while also addressing issues related to ice crystal formation and cell wall rupture during freezing [26,27].

In modern bread production, hydrocolloids are thus highly valued for their textural and stabilizing properties. Regardless of the origin of hydrocolloids, being secreted by plants (e.g., guar gum), produced by micro-organisms (e.g., xanthan gum), or plant materials modified by chemical and physical methods (e.g., cellulose derivatives) [24], these biopolymers are all classified as food additives under the provisions of the food law and have E numbers under EU Regulation (no. 1333/2008) [28]. However, due to regulatory requirements, such as those in the EU where hydrocolloids must be labeled as functional ingredients with E numbers, there is a growing trend to bypass these additives (clean label) [29,30]. Instead, natural ingredients like psyllium husk and inulin are increasingly used, especially in gluten-free formulations [31,32,33]. These ingredients strongly contribute to the functionality of the dough, without having to be labeled with an E number. This shift caters to the growing consumer demand for cleaner labels and healthier bread options while maintaining the desired functional properties. Consequently, the integration of diverse natural fibers into bread formulations is becoming increasingly relevant.

Although the use of dietary fibers from fruits and vegetables in bread products is not commercially widespread, fiber enrichment provides health benefits, manages by-products from fruit and vegetable processing, and can improve company performance [34]. Innovation in fiber-enriched products is an important competitive tool, as it helps maintain consumer interest by expanding the range of bread products with increased nutritional value. Furthermore, in specialty bread products, legume flours are often added for their functional components, alongside their high protein content. Seeing an opportunity to enhance the functionality of commercial wheat-based bread types and recognizing the increased economic interest in incorporating dietary fibers, this study aimed to evaluate the influence of different types of dietary fibers on the properties of wheat-based dough and bread using rheological methods and baking trials. Specifically, pea, cocoa, and apple fiber were selected to represent a diverse range of natural fibers from different origins. This selection was intended to provide a comprehensive analysis of how various types of fiber affect bread properties. Additionally, this study seeks to establish guidelines for testing new fibers in bread and to prioritize the rheological methods necessary to elucidate the behavior of dietary fibers in wheat-based dough systems.

## 2. Materials and Methods

### 2.1. Materials

A commercial wheat flour (Epi B type 55) supplied by Paniflower (Merksem, Belgium) was used for all the experiments in this study. The flour met following specifications: max. 15.5 g of water/100 g of flour, 12–13 g of protein/100 g of flour, and max. 0.68 g of ash/100 g of flour. The pea fiber (PF) “Emfibre EF200” was kindly donated by Emsland Group (Emlichheim, Germany), as were the cocoa (CF) and apple (AF) fiber “m20” by Greenfield (Warsaw, Poland). The PF, CF, or AF were each blended into the wheat flour at replacement levels of 0, 1, 5, and 10 g of fiber powder/100 g of wheat flour (*w*/*w*%). Other ingredients such as dried yeast and table salt were obtained from the local market.

### 2.2. Flour and Dough Characteristics

#### 2.2.1. Water Retention Capacity

To determine the water retention capacity (WRC), an adapted method based on the standard AACC method 56-11 (AACC, 2000) for the determination of the solvent retention capacity profile was used. A sample of 1.000 ± 0.050× *g* of the flour/fiber mixture was accurately weighed into a 15 mL centrifuge tube. After the addition of 10 mL deionized water, the tube was shaken vigorously to create a homogeneous suspension. Following a 30 min solvating and swelling period, the tube was centrifuged at 4000× *g* for 15 min. After decanting and 10 min of draining, the tube was reweighed.

#### 2.2.2. Water Absorption and Kneading Properties

A farinograph mounted with a 50 g mixing bowl (Farinograph-E, Brabender GmbH, Duisburg, Germany) was used to determine the effect of fiber incorporation on the water absorption and kneading properties of wheat dough according to ICC standard method no. 115/1. A consistency of 500 farinograph units (FU) was aimed for by adapting the water absorption. Different dough parameters, including water absorption (WA, %), dough development time (DDT, min, time for the dough to reach gluten hydration peak), stability (STAB, min, time consistency remains constant at max.), and softening (SOFT, FU, consistency difference 12 min after DDT), can be derived from the resulting farinograms, which plot consistency (FU) in the function of time.

#### 2.2.3. Chopin Alveograph

The Alveograph NG (Chopin Technologies, Villeneuve-en-Garenne, France) was used to evaluate the viscoelastic attributes at farinograph absorption with the standard AACC method 54-30 (AACC, 2000). The dough tenacity (P), extensibility (L), and deformation energy (W) were registered by the Alveolink NG software program.

#### 2.2.4. Pasting Properties

To determine the pasting properties of the samples, a Rheometer MCR 102 equipped with a starch gelatinization cell with stirrer ST24-2D and software ReoCompass 1.16 (Anton Paar GmbH, Graz, Austria) was used. Based on the moisture content of the sample, the correct amount was weighed and dispensed in 20 mL of distilled water (e.g., for 14 g water/100 g flour, 2.8 g sample was added). Prior to the shear phase, the suspension was heated to 50 °C and simultaneously stirred at 960 rpm to obtain a homogeneous dispersion of the sample. The rotation speed was lowered to 160 rpm for the remainder of the test. After holding the temperature at 50 °C for 1 min, it was raised to 95 °C at a constant rate of 5 °C/min. Subsequently, the temperature was held at 95 °C for 5 min and afterward cooled to 50 °C at the same rate as the temperature increase. Finally, it was held at 50 °C for 2 min. This procedure [35] was adapted from the AACC International Method 76-21-02 [36], yielding the following parameters derived from the gelatinization curves: initial viscosity (IV), pasting temperature (T_past_), peak viscosity (V_peak_), peak temperature (T_peak_), holding strength (HS), breakdown (BD), final viscosity (V_final_), setback from peak (SB_peak_), and total setback (SB_total_).

#### 2.2.5. Dough Expansion during Proofing (Yeast Activity and Dough Rise)

A small amount of dough was prepared in the 50 g mixing bowl of the farinograph using Epi B flour with 0, 1, 5, or 10% fiber replacement. For 50 g mixture, 1.25 mg of vitamin C, 0.5 g of dry yeast, and 0.05 g of malt flour were also added. Farinograph water absorption was used to determine the amount of water (with 0.75 g of table salt) to be added. Dough expansion during proofing was evaluated with a TA.XT*plus* Texture Analyzer (software Exponent 6.1.7.0; load cell 5 kg; Stable Micro Systems, Godalming, UK). The applied method was similar to the one described by Debonne et al. [37], where a cylindrical cup (inner ⌀ 5 cm, height 10.5 cm) and accompanying fitting probe (⌀ 5 cm) were used. The probe registered the force of the rising dough for 3600 s; it was kept at a constant temperature of 30 (±0.5) °C to simulate a proofing cabinet. The following settings were applied: test speed 2.00 mm/s and force 301 g, while proofing height (PH) (mm) was collected from the data as parameter.

### 2.3. Bread-Making Procedure

The bread-making procedure is based on the techniques described by Debonne et al. [38] and begins by preparing a dough in which 0, 1, or 5% of the flour is replaced by fiber. The exact composition of each formulation is presented in Table 1. After kneading in a De Danieli spiral mixer for 7 min, the dough was proofed (Panimatic, Souppes-sur-Loing, France) for 10 min at 30 °C and 80–90% relative humidity (RH). The dough was divided into 70 g loaves, manually rounded, and fermented again on a perforated, greased plate for 60 min (30 °C, 80–90% RH). The loaves were then placed in the oven (MIWE aeromat FB12, Arnstein, Germany) and baked in two phases. The first step involved an oven temperature of 220 °C for 2 min and a steam addition of 200 mL with a closed steam valve, followed by a 13 min second phase with an oven temperature of 200 °C, no steam addition, and an open steam valve. After baking, the loaves were cooled at room temperature for 2 h prior to evaluative analysis.

### 2.4. Technological Bread Evaluation

The bread loaves were weighed on a KERN balance (±0.01 g) (Balingen, Germany). Weight loss due to baking was calculated from comparison with the weight before baking. A Volscan Profiler 600 (Stable Micro Systems, Godalming, UK) was used to measure the volume of the loaves, and the color parameters (L, a, b) of both the crust and crumb were quantified with a CM700d/600d spectrophotometer (Konica Minolta, Tokyo, Japan), after standardization with a calibration plate. While the crumb moisture content (MC) was determined according to the AACC International Method (44-15.02) [39], its texture was evaluated with a texture profile analysis (TPA). Hardness (HARD), springiness, (SPRING), cohesion (COH), chewiness (CHEW), and resilience (RES) were defined using two compressions (40% strain, test speed 1.7 mm/s) of a cylindrical probe made of plexiglass with a diameter of 25 mm (P/25P) on three stacked slices (thickness of 9 mm) of bread.

### 2.5. Statistical Analysis

All experiments were conducted in triplicate at least, and the results were reported as the mean ± standard deviation. Data analysis was carried out with SPSS Statistics 27 (SPSS Inc., Chicago, IL, USA). The prerequisite of normality was not met for each parameter; therefore, the data was submitted to a Kruskal–Wallis test with subsequent pairwise comparison to assess significant differences between different substitution levels (*p* < 0.05). The principal component analysis (PCA) was carried out in R (Version 4.3.1) using the built-in prcomp function [40]. The graphical representation of the PCA results was generated with the factoextra and ggplot2 packages [41,42], while other visualizations were created using SigmaPlot (Version 15, Inpixon, Palo Alto, CA, USA).

## 3. Results and Discussion

### 3.1. Compositional Information Regarding Featured Fiber Products

There is a wide variety in the composition of commercially available fibers, mainly due to their origin, production methods, extraction processes, and milling techniques, resulting in diverse physicochemical properties [43]. Because it is complex, expensive, and time-consuming to perform a full compositional characterization in application-oriented research and industry, this process was bypassed in this study. The aim of this study was to gain more insight into the general effects of fibers on dough and bread quality. To attribute certain effects and observed characteristics, available information on the dietary fiber composition of the featured fiber products was collected and is presented in Table 2.

The pea fiber originates from the cotyledon of yellow peas, also known as the “inner fibers”, which are largely composed of non-starch polysaccharides, such as hemicellulose, pectin, and gums [44,45]. Pfoertner and Fischer [44] also noted that commercially available pea cotyledon fibers contain high concentrations of starch, which can also contribute to their specific physicochemical properties (e.g., the formation of gelled structures when heated above gelation temperature). The apple fiber is obtained from dried apple pomace. The insoluble dietary fiber (IDF) predominantly consists of cellulose with smaller amounts of lignin and hemicellulose, while the soluble dietary fiber (SDF) is mainly composed of pectin [46]. The cocoa fiber is produced by micronizing cocoa shells. The IDF consists mainly of cellulose, with hemicelluloses and pectic substances associated with the cell wall matrix also present. The SDF fraction is primarily composed of pectin, with minor amounts of galactomannans [48,49].

It should be noted that commercially available fiber ingredients, even though they have a high concentration of fiber, are not purified or concentrated and therefore also contain other components. For example, the technical datasheet of the PF reports 17% carbohydrates, indicating the presence of starch, and 11% protein. Additionally, the nutritional values of AF report relatively high concentrations of carbohydrates, including sugars, while CF contains a high concentration of protein and minerals. These other remaining components can also impact the dough and pasting behavior. As previously mentioned, this paper adopts a more integrated approach to understanding the effects of incorporating these ingredients into bread, rather than a full-scale compositional analysis.

### 3.2. Effect of Fiber Concentration (0–10%) on Dough and Gelatinization Parameters

#### 3.2.1. Traditional Dough Analysis

**Figure 1:**
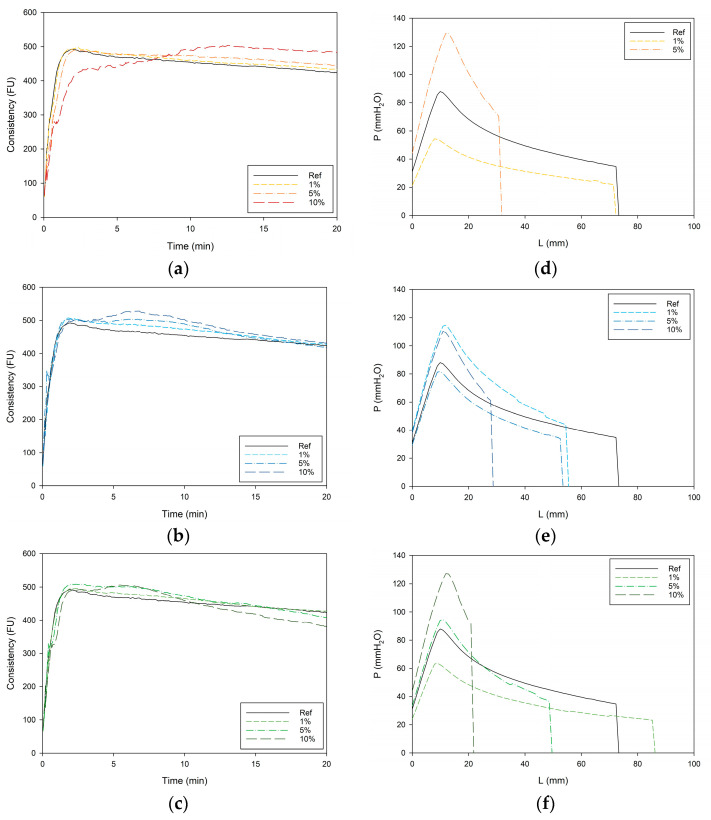
Farinogram (**a**–**c**) and alveogram (**d**–**f**) profiles as influenced by the incorporation of pea (**a**,**d**), cocoa (**b**,**e**), and apple (**c**,**f**) fibers in concentrations of 0 (Ref), 1, 5, and 10% (or g/100 g wheat flour) (n = 3).

The farinograph analyses show the great impact of increased fiber replacement on the farinogram profiles. It can be observed in Figure 1 that for each type of fiber, next to the first peak, a second peak emerges from the farinogram as the fiber concentration is increased.

As previously reported in literature, farinograms of fiber-supplemented dough display two peaks [15,50,51,52,53]. The first peak can be attributed to the hydration of gluten, while the second peak corresponds to the hydration of the fiber components. As fibers exhibit greater water absorption than gluten, water migration can occur during mixing. This involves partial dehydration of the flour components while the fiber components are hydrated [50]. The reduced availability of water for the gluten network causes conformational changes in the gluten proteins, making the dough stiffer and more resistant to mixing, thereby increasing consistency [50,54,55].

At low fiber concentrations, the second peak is difficult to distinguish and could be misconstrued as increased dough stability. Conversely, in formulations with high fiber concentrations, the second peak is sometimes used prematurely to determine the DDT. This suggests that, although the farinogram shapes provide valuable insights into the kneading process, standard farinographic parameters are not suitable for drawing conclusions about the dough quality of wheat-based doughs with fiber. The competition for water and the resulting stiffening of the dough is also evident in the alveograph curves (Figure 1d–f), leading to decreased extensibility (L) and increased tenacity (P). This effect is also observed in the proofing experiment (Supplementary Materials Table S1), where an increased fiber concentration significantly impacts the proofing capacity negatively. However, it is challenging to precisely attribute the extent of these effects to specific fiber components. Interestingly, AF in low concentrations slightly improves the proofing height, possibly because the available sugars serve as a substrate for yeast.

With increased fiber concentration, the WA of the dough also increased. Compared to the reference dough (WA: 59.9 ± 0.0 g/100 g flour), the WA value increased by 4.5, 12, and 22% for 1, 5, and 10 g of pea fiber substitution per 100 g of wheat flour, respectively. Similar increases (3.2, 11, and 23%) were observed for apple fiber. While the WA increase for cocoa fiber was still significant, it was somewhat lower, at 1.8, 8.3, and 13% for the same levels of substitution. These WA increases can be attributed to the high water-holding capacity of the fiber fractions [56,57].

#### 3.2.2. Pasting Analysis

**Figure 2:**
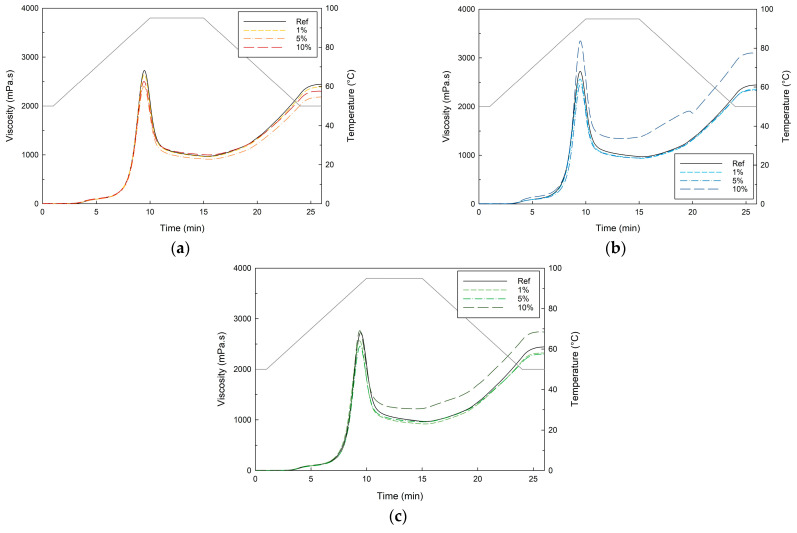
Pasting behavior of pea (**a**), cocoa (**b**), and apple (**c**) fiber/wheat flour mixtures in concentrations of 0 (Ref), 1, 5, and 10% of fiber (or g/100 g of wheat flour) (n = 3).

As the farinograph and alveograph measurements primarily reflect the impact of fiber components on the development of the gluten network, the pasting properties can provide more information on the gelling properties of the fiber components as well as possible interactions with wheat starch. Figure 2 presents the gelatinization curves of the fiber/wheat flour mixtures. Whereas PF incorporation and low concentrations of AF and CF exhibit only limited effect on the pasting properties, a high concentration of AF and CF increases the stability (increased HS) and final viscosity, indicating these ingredients contain gel-forming fibers, such as pectins and galactomannans. It is worth noting that pectins from different sources can behave differently, which may also contribute to the observed variations in the pasting properties [58].

#### 3.2.3. Cluster Analysis

**Figure 3:**
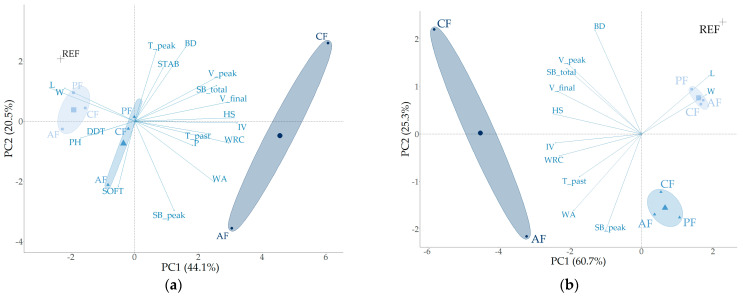
Principal component analysis of dough and gelatinization parameters based on wheat flour/fiber mixtures of 0% (REF, +), 1% (■), 5% (▲), and 10% (●) (excluding 10% pea fiber) with pea fiber (PF), apple fiber (AF), and cocoa fiber (CF): (**a**) inclusion of all parameters, (**b**) optimized PCA. Parameter abbreviations are defined in Section 2.

The comprehensive analysis of wheat-based dough and bread incorporating pea, apple, and cocoa fiber yielded an extensive dataset (Supplementary Materials Tables S1–S4). A PCA was performed based on the dough and gelatinization parameters of the wheat-based flour/fiber mixtures, explaining 64.6% of the total variance (PC1: 44.1%; PC2: 20.5%), as shown in Figure 3a. No strong correlations (r < 0.58) were observed between DDT, STAB, and SOFT with any of the other parameters. The informative content of the PCA plot using the dough and gelatinization properties was optimized by maximizing the sum of variances in dimensions 1 and 2. The optimization was achieved by excluding several parameters such as PH, P, T_peak_, and certain farinographic data (STAB, SOFT, and DDT) from the final analysis.

Based on the resulting optimal PCA plot (Figure 3b), where the first two dimensions account for 86.0% of the total variance, and the observation that the samples are clustered by fiber concentration, several interpretations and practical insights can be derived for optimizing dough and bread production with varying fiber concentrations. Regardless of the type of fiber, the samples with 1% and 5% fiber concentrations tend to cluster together. This implies similar water absorption and dough characteristics within these concentrations, suggesting that low fiber replacements have a consistent and predictable effect on the dough and gelling properties. Conversely, samples with 10% fiber exhibit greater variability (see AF10 and CF10, with PF10 excluded in Figure 3 due to missing alveograph data for PF10). This higher variability indicates that high fiber replacements introduce more complexity in the dough and gelatinization properties.

**Figure 4:**
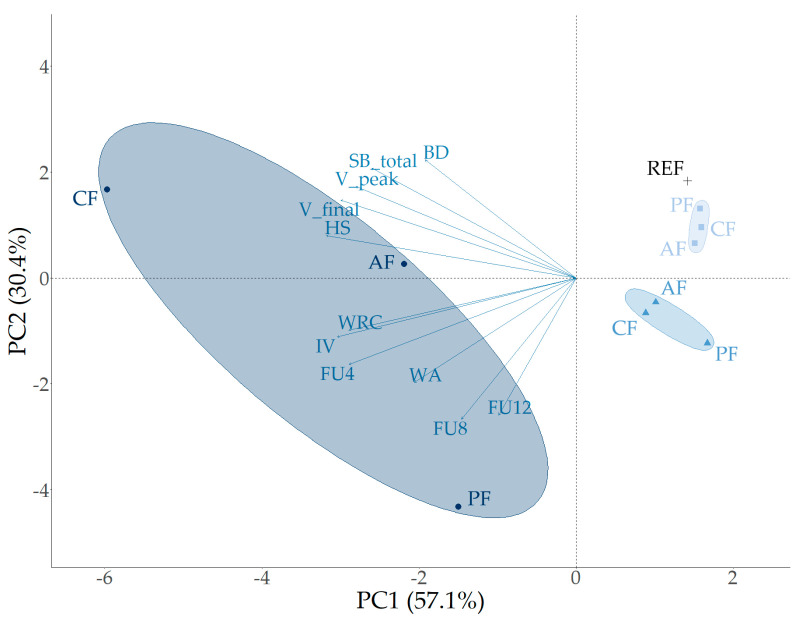
Principal component analysis of dough and gelatinization parameters based on wheat flour/fiber mixtures of 0% (REF, +), 1% (■), 5% (▲), and 10% (●) with pea fiber (PF), apple fiber (AF), and cocoa fiber (CF): optimized PCA plot with inclusion of new farinographic parameters (FU_4_, FU_8_, and FU_12_). Parameter abbreviations are defined in Section 2.

The altered shape of the farinograms with high fiber concentration complicates the interpretation of the software-generated parameters (DDT, STAB, and SOFT). The standard method cannot account for the second shoulder. Traditionally, SOFT is measured 12 min after reaching maximal peak consistency. Alternatively, the degree of softening can be recorded over a span of 12 min after the first peak, with measurements taken at 4, 6, 8, 10, and 12 min (denoted as FU_x_, where x is the time in minutes after the first peak, and FU represents the dough consistency (expressed in farinograph units, FU) relative to the consistency of the first peak, in %). The presence of a second shoulder within these intervals is reflected in the results (e.g., FU_x_ > 100%). When excluding the original farinographic data (SOFT, STAB, and DDT), while incorporating the new parameters (FU_4_, FU_6_, FU_8_, FU_10_, and FU_12_) in an unoptimized PCA, the sum of the dimensions PC1 and PC2 accounted for 67.1% of the total variance. However, after optimization—removing parameters PH, T_peak_, T_pasting_, SB_peak_, P, W, L, FU_6_, and FU_10_—the sum of the dimensions PC1 and PC2 increased to 87.5% (Figure 4).

The better observed fit when the new parameters (FU_4_, FU_8_, and FU_12_) were used, as indicated by the increase in the sum of dimensions (PC1 + PC2 = 87.5%), can be attributed to several key factors. First, the standard farinographic parameters were not designed to capture the complex hydration behavior of fibers at high concentrations. Fibers have unique hydration properties that can create a second shoulder in the farinogram, a phenomenon not adequately represented by traditional parameters. The new parameters capture these specific hydration dynamics better, providing a more accurate representation of the rheological properties of the dough under high fiber conditions. Second, by removing parameters that may not be as relevant to the behavior of fiber-enriched dough, the model avoids noise and irrelevant variability. This exclusion helps in refining the model to focus on the most significant factors influencing dough behavior at high fiber concentrations. Third, the process of optimization itself, which includes the selection and incorporation of FU_4_, FU_8_, and FU_12_ while excluding less relevant parameters, enhances the ability of the model to explain the variance. This suggests the new parameters are more aligned with the principal components of the data, resulting in a higher percentage of the total variance being explained by the first two principal components.

### 3.3. Effect of Concentration (0–5%) and Type of Fiber on Dough and Bread Quality

In this study, no changes were made to the dough preparation for baking, which led to the absence of the results of bread with high fiber concentrations (10%). On the contrary, for high fiber concentrations (10%), the greater variability suggests that more careful monitoring and adjustments in production are needed to achieve consistent bread quality. This may involve more frequent testing and fine-tuning of the recipe and process parameters, including the new farinographic parameters.

**Figure 5:**
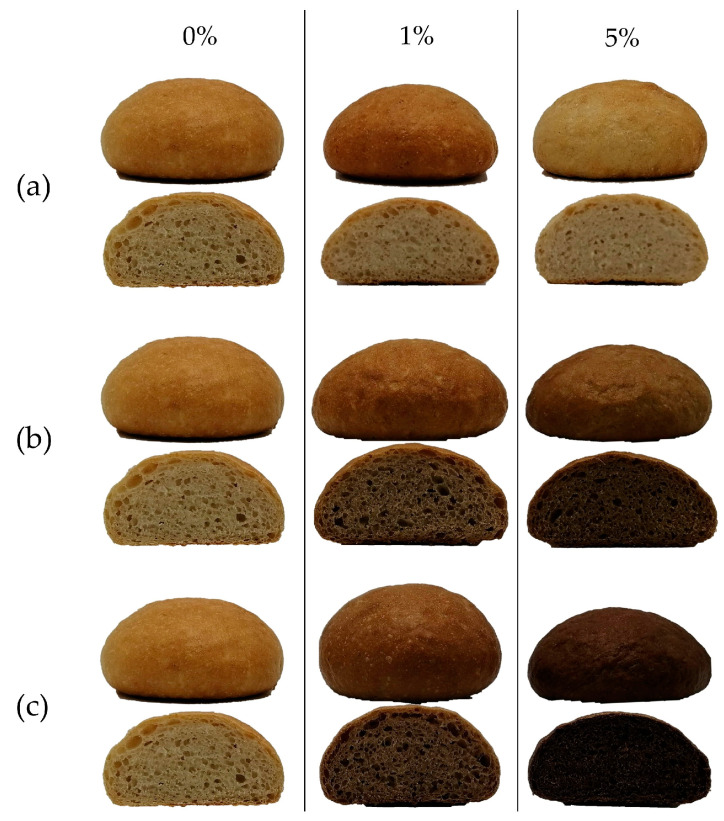
Wheat-based bread containing 0%, 1%, and 5% pea fiber (**a**), cocoa fiber (**b**), or apple fiber (**c**).

For dough and bread formulations up to 5% fiber inclusion, traditional dough analysis parameters remained applicable. Moreover, a 5% fiber inclusion is beneficial from a marketing perspective as the threshold for the ‘source of fiber’ claim (≥3%) is met for each of the types of fiber (Table 3) [59]. The impact of incorporating fiber into wheat flour on the resulting bread is illustrated in Figure 5. It mainly affects the lightness (L-value) of the bread, with a slight increase observed for PF, whereas the bread containing AF and CF is significantly darker (Supplementary Materials Table S3).

**Figure 6:**
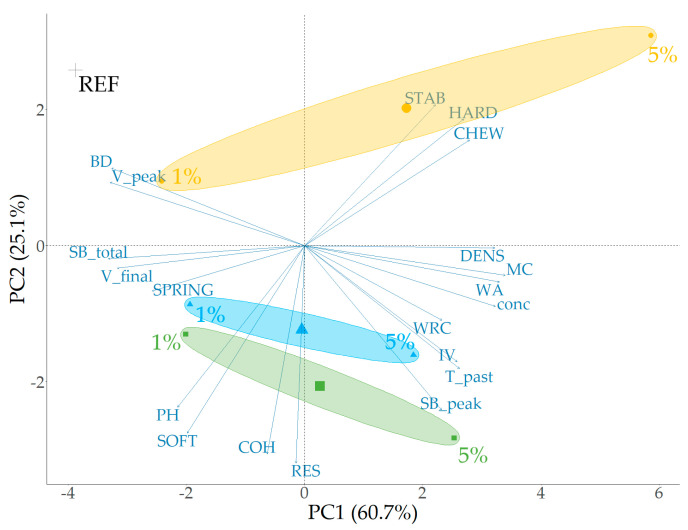
Principal component analysis obtained from the quality parameters of wheat-based dough and bread (+) with pea (●, yellow), apple (■, green), or cocoa (▲, blue) fiber incorporation—data of REF, 1%, and 5% combined. Parameter abbreviations are defined in Section 2.

Based on the results of the wheat-based fiber-incorporated dough and bread quality parameters, another optimized PCA was conducted. The analysis was optimized by maximizing the sum of the variances in dimensions 1 and 2 of the PCA. This led to the exclusion of specific input variables such as the alveograph parameters (P, L, and W scores), certain gelatinization parameters (T_peak_ and HS) obtained from the rheometer analysis, and the DDT of the farinograph measurement. As an alveograph primarily measures parameters related to gluten strength and dough extensibility, these parameters do not fully capture the behavior of doughs with non-gluten ingredients like fibers, which have different hydration and structural properties [60]. Furthermore, a key constraint in this optimization was the inclusion of all measured baking quality parameters to accurately depict the relationship between the dough characteristics and final product attributes. The difference between the standard and new farinograph parameters for low fiber levels (up to 5%) was substantial enough to justify using the standard farinograph parameters, respectively, the sum of PCA dimensions 1 and 2 for scenario 1 is 85.8%, and for scenario 2 is 79.4%. Figure 6 shows the final optimized PCA plot, which demonstrates clustering for the PF, CF, and AF, indicating that the type of fiber and its concentration (0%, 1%, or 5%) contribute significantly to the observed variation.

Based on these results, the primary texture attributes of wheat-based breads containing 0, 1, and 5% fiber, including crumb hardness, chewiness, and springiness, exhibit a strong correlation with fiber concentration as well as water-related and starch-based properties. An increase in fiber concentration is associated with higher MC, WA, and density. These parameters show a positive correlation with both hardness and chewiness (r ≥ 0.70). The high hydration properties of fiber lead to increased WA and MC in the final bread product, resulting in a denser crumb structure that contributes to greater hardness and chewiness. Additionally, the retained water can influence the gelatinization and retrogradation of starch. During baking, starch granules absorb water, swell, and gelatinize, which contributes to the structure of the bread [61]. Fiber can interfere with this process by competing for water, leading to less complete gelatinization [62]. A concentration effect was observed for the rheological parameters V_peak_, SB_total_, and V_final_, which decreased with increasing fiber concentration. These parameters were negatively correlated with hardness (r ≤ −0.71) and chewiness (r ≤ −0.73) and positively correlated with springiness (r ≥ 0.72). The negative correlation between SB_total_, V_final_, and hardness (and chewiness) indicates that lower values of these viscosity parameters (with increased fiber content) result in a harder and chewier crumb. The positive correlation with springiness suggests that reduced retrogradation (lower SB_total_) results in a more elastic and springier crumb. While fiber concentration significantly affects hydration and structural properties, it does not impact crumb cohesion (the ability of the crumb to stay together) or resilience (the ability of the crumb to recover its shape after compression). These properties are more influenced by the protein network (gluten), the presence of other structural components, and the overall dough structure rather than just the fiber content.

## 4. Conclusions

The use of fiber-rich ingredients in bread products offers several benefits, including health advantages, managing by-products from fruit and vegetable processing, and expanding the product range for producers. One key advantage of fiber incorporation is its high water-absorption capacity, which increased by 12.0%, 8.3%, and 10.9% in doughs containing 5% pea, cocoa, or apple fiber, respectively. This increase contributed to an augmented crumb moisture content in the resulting baked bread (+6.0%, +4.4%, and +4.7%), thereby extending the physicochemical shelf-life of the product. However, the addition of fiber often impacts bread density, texture, and sensory quality. In bread with 5% pea fiber, density and hardness increased up to 25% and 50%, respectively. In contrast, the increase in these parameters was limited for 5% apple fiber (+20% and +13%), and even beneficial for 5% cocoa fiber, with a 4.4% decrease in hardness. Yet, the crust and crumb lightness significantly deviated for bread with 5% cocoa fiber (−35.3% and −45.6%) and 5% apple fiber (−13.5% and −19.8%). Differences in the origin of the fiber-rich materials, as well as in production and pre-treatment strategies, strongly impact the physicochemical properties and application possibilities of the fibers. This study evaluated the effects of fiber concentration (0–10%) and type (apple, cocoa, or pea) on dough and bread quality using optimized principal component analysis (PCA).

In optimizing the PCA, traditional alveograph parameters (P, L, and W) and certain farinograph and rheometer parameters (DDT, T_peak_, and HS) were excluded due to their inadequacy in capturing the hydration and structural dynamics of fiber-rich doughs. A second shoulder in the farinogram, caused by fiber hydration, necessitated new parameters that better captured the dynamics of high-fiber doughs. Excluding less relevant traditional parameters reduced noise and variability, focusing on significant factors influencing high-fiber dough behavior. New farinographic parameters (FU_4_, FU_6_, FU_8_, FU_10_, and FU_12_), as well as the optimization of the PCA, significantly improved the explanatory power of the model, increasing the sum of variances in PCA dimensions 1 and 2 from 67.1% to 87.8%. These new parameters provided a more accurate representation of dough rheology under high-fiber conditions and could prove useful in fine-tuning process parameters in these types of doughs.

At low fiber concentrations (up to 4 g fiber/100 g bread), the negative effects on dough rheology and bread quality were minimal. This study showed that fiber levels up to 5% of the final dough weight (by flour replacement) affected the dough and bread irrespective of fiber type. This result suggests that there is no need for a comprehensive compositional analysis at low levels of fiber. Traditional dough analysis techniques, which cover starch and protein behavior, partially capture fiber effects but are inadequate at higher fiber levels. High fiber concentrations (namely 10%) showed increased variability and complexity in the dough properties. Distinct clustering in the PCA biplot indicates significant impacts from both fiber concentration and type. Low fiber concentrations (1–5%) had consistent and predictable effects.

In addition to nutritional benefits, production feasibility, and end product quality, it is important to consider potential market introduction. Incorporating fiber at certain percentages could influence production cost and final product pricing. For instance, fibers with high water absorption can increase water usage and decrease the amount of higher-cost ingredients in the recipes. Additionally, the use of by-products from fruit and vegetable processing can be more economical than refined additives. Furthermore, marketing health claims, such as ‘source of fiber’, can attract health-conscious consumers and potentially justify higher product pricing.

In summary, optimizing the rheological parameters for fiber-augmented flour is crucial for adapting the bread-making process to account for the impact of fiber on dough properties. This leads to high-quality bread with the desired characteristics and enhanced nutritional benefits, as well as potential economic advantages.

## Figures and Tables

**Table 1:** Formulations (expressed as percentages) of wheat-based dough in which 0, 1, or 5% of the flour is replaced by pea, apple, or cocoa fiber.

	**REF**	**Pea**		**Apple**		**Cocoa**	
		1%	5%	1%	5%	1%	5%
Flour/Fiber *	60.8	59.8	58.0	60.3	58.8	60.1	58.3
Flour	60.8	59.2	55.1	59.7	55.9	59.5	55.4
Fiber	0	0.60	2.90	0.60	2.94	0.60	2.92
Yeast	0.63	0.62	0.60	0.62	0.61	0.62	0.60
Malt	0.06	0.06	0.06	0.06	0.06	0.06	0.06
Salt	0.94	0.92	0.90	0.94	0.91	0.93	0.91
Vitamin C	0.0016	0.0015	0.0015	0.0016	0.0015	0.0015	0.0015
Water **	37.6	38.6	40.4	38.1	39.6	38.3	40.1

* The amount of flour/fiber mixture was corrected to obtain 14% moisture content. ** The added amount of water for each individual recipe depended on the water absorption recorded with the farinograph.

**Table 2:** Data from the literature and information provided by the supplier regarding the total dietary fiber (TDF), insoluble dietary fiber (IDF), and soluble dietary fiber (SDF) content of the pea (PF), apple (AF), and cocoa (CF) fibers (g/100 g dm).

	Supplier	Literature	
Pea		[44]	[45]
Total DF	Approx. 65	60.0	76.3
Apple		[46]	[47]
Total DF	51.0–60.0	51.1	82.0
Insoluble DF	47.0–52.0	36.5	77.8
Soluble DF	4.0–8.0	14.6	4.2
Cocoa		[48]	[49]
Total DF	56.0–62.0	28.1 ^a^ (60.5 *)	35.7 ^a^ (63.6 *)
Insoluble DF	47.0–50.0	18.0 ^a^ (50.4 *)	24.0 ^a^ (51.9 *)
Soluble DF	9.0–12.0	10.1	11.7

^a^ calculated * This value includes the Klason lignin. In some plants, this fraction represents lignin, while in others, it includes tannin/protein complexes and Maillard products from roasting. Therefore, in products such as cocoa that contain these complexes, the determination of Klason lignin is not representative of the actual amount of lignin.

**Table 3:** Calculated nutritional content of wheat-based breads with 0% (Ref), 1%, or 5% pea fiber (PF), cocoa fiber (CF), and apple fiber (AF). Nutritional values of the ingredients and baking loss were taken into account.

Per 100 g	0% REF	1%			5%		
		PF	CF	AF	PF	CF	AF
Energy (kJ)	1024	1023	1004	1000	944	947	953
Energy (kcal)	252	251	247	246	233	233	235
Fats (%)	0.9	0.9	0.9	0.9	0.8	0.9	0.9
Saturates (%)	0.2	0.2	0.2	0.2	0.2	0.3	0.2
Carbohydrates (%)	49.1	48.8	48.0	47.7	44.3	44.1	44.9
Sugars (%)	1.0	1.0	1.0	1.1	1.0	1.0	1.3–1.6
Protein (%)	8.6	8.6	8.5	8.4	8.0	8.2–8.5	7.8–8.0
Salt (%)	1.1	1.1	1.1	1.1	1.1	1.1	1.1
Fibers (%)	2.3	2.7	2.7	2.6–2.7	4.1	4.0–4.2	3.8–4.1

## Data Availability

The original contributions presented in the study are included in the article/Supplementary Material, further inquiries can be directed to the corresponding author.

## Supplementary Materials

The following supporting information can be downloaded at: https://www.mdpi.com/article/10.3390/foods13162582/s1, Table S1: Impact of pea (PF), cocoa (CF), and apple (AF) fibers on water retention capacity (WRC) and dough properties of fiber/wheat flour mixtures: farinograph parameters [water absorption (WA), dough development time (DDT), dough stability (STAB), and dough softening (SOFT)]; alveograph parameters [dough tenacity (P), dough extensibility (L), and deformation energy (W)]; and proofing [proofing height (PH)] (n = 3); Table S2: Impact of pea (PF), cocoa (CF), and apple (AF) fibers on pasting properties of fiber/wheat flour mixtures: initial viscosity (IV), pasting temperature (T_past_), peak viscosity (V_peak_), peak temperature (T_peak_), holding strength (HS), final viscosity (final), breakdown (BD), setback from peak (SBp_eak_), total setback (SB_total_) (n = 3); Table S3: Impact of pea (PF), cocoa (CF), and apple (AF) fibers on bread properties: moisture, baking loss, density, and color parameters of the crust and crumb [L, a, b] (n = 3); Table S4: Impact of pea (PF), cocoa (CF), and apple (AF) fibers on texture profile analysis (TPA) parameters [hardness, cohesiveness, chewiness, springiness, and resilience] (n = 3).
