# Supplementary material for: Technological Evaluation of Fiber Effects in Wheat-Based Dough and Bread
Source: Foods. 2024 Aug 18;13(16):2582. doi: 10.3390/foods13162582 (PMC11353414; doi:10.3390/foods13162582)
Supplement: Supplementary file 1 [file foods-13-02582-s001.zip › foods-3134330-supplementary.pdf]

foods

MDPI

Supplementary Materials

Table S1. Impact of pea (PF), cocoa (CF), and apple (AF) fiber on water retention capacity (WRC) and dough properties of fiber/wheat flour mixtures: farinograph parameters [water absorption (WA), dough development time (DDT), dough stability (STAB), and dough softening (SOFT)], alveograph parameters [dough tenacity (P), dough extensibility (L), and deformation energy (W)], and proving [proving height (PH)] (n=3).

			Farinograph				Alveograph			Proving
	Fiber conc. (g/100 g flour)	WRC (%)	WA (%)	DDT (min)	STAB (min)	SOFT (FU)	P (mm H_2O)	L (mm)	W (10^{-4} J)	PH (mm)
PF	0	68.3 ± 1.2^{ab}	59.9 ± 0.0^{a}	2.1 ± 0.1^{a}	9.2 ± 1.1^{ac}	39.7 ± 5.9^{ad}	97 ± 1^{acd}	67 ± 6^{a}	240 ± 13^{a}	49.9 ± 0.2^{ade}
	1	65.3 ± 0.7^{a}	62.6 ± 0.0^{ab}	2.3 ± 0.1^{ac}	9.2 ± 1.1^{ac}	37.7 ± 5.0^{ad}	60 ± 2^{a}	67 ± 4^{a}	152 ± 10^{b}	47.1 ± 0.5^{ab}
	5	72.9 ± 0.4^{bcd}	67.1 ± 0.4^{bce}	2.8 ± 0.9^{ac}	13.2 ± 1.5^{ab}	19.3 ± 6.8^{bc}	143 ± 2^{b}	29 ± 3^{bc}	180 ± 15^{ab}	38.3 ± 1.0^{bc}
	10	81.3 ± 0.4^{cd}	73.0 ± 0.0^{ce}	12.2 ± 0.6^{b}	17.2 ± 0.2^{b}	14.3 ± 2.9^{b}	*	*	*	24.6 ± 0.7^{c}
CF	0	68.3 ± 1.2^{ab}	59.9 ± 0.0^{a}	2.1 ± 0.1^{a}	9.2 ± 1.1^{ac}	39.7 ± 5.9^{ad}	97 ± 1^{acd}	67 ± 6^{a}	240 ± 13^{a}	49.9 ± 0.2^{ade}
	1	64.1 ± 2.8^{a}	61.0 ± 0.0^{ad}	2.2 ± 0.3^{ad}	10.5 ± 1.3^{ab}	30.7 ± 7.5^{abd}	127 ± 13^{bc}	46 ± 9^{abc}	234 ± 11^{a}	50.6 ± 1.0^{ad}
	5	70.6 ± 1.0^{ac}	64.9 ± 0.1^{ac}	1.9 ± 0.4^{a}	10.6 ± 0.3^{ab}	17.7 ± 0.6^{bc}	90 ± 2^{ac}	51 ± 3^{ab}	177 ± 10^{ab}	46.8 ± 0.9^{ab}
	10	87.9 ± 3.3^{d}	67.9 ± 0.9^{bce}	6.7 ± 0.4^{bc}	9.4 ± 0.5^{ac}	22.7 ± 2.3^{ab}	122 ± 3^{bc}	26 ± 3^{bc}	139 ± 7^{b}	43.7 ± 0.3^{bc}
AF	0	68.3 ± 1.2^{ab}	59.9 ± 0.0^{a}	2.1 ± 0.1^{a}	9.2 ± 1.1^{ac}	39.7 ± 5.9^{ad}	97 ± 1^{acd}	67 ± 6^{a}	240 ± 13^{a}	49.9 ± 0.2^{ade}
	1	70.2 ± 1.5^{ac}	61.8 ± 0.0^{ab}	2.4 ± 0.1^{ab}	9.9 ± 0.3^{abc}	32.7 ± 1.5^{acd}	70 ± 3^{a}	71 ± 15^{a}	179 ± 22^{ab}	52.0 ± 0.7^{ade}
	5	78.9 ± 13.6^{bcd}	66.4 ± 0.1^{bcde}	2.4 ± 0.5^{ad}	7.7 ± 0.2^{c}	39.3 ± 5.9^{ad}	104 ± 5^{ab}	43 ± 7^{abc}	177 ± 17^{ab}	53.1 ± 0.5^{de}
	10	82.4 ± 3.7^{cd}	73.8 ± 0.2^{e}	5.6 ± 0.1^{bcd}	6.8 ± 0.6^{c}	48.7 ± 3.1^{d}	140 ± 7^{bd}	21 ± 0^{c}	133 ± 7^{b}	45.7 ± 1.9^{bce}

[a-e] Results marked by the same letter within the same column are not statistically different ($p > 0.05$).

* Measurement not possible.

foods MDPI

Table S2. Impact of pea (PF), cocoa (CF), and apple (AF) fiber on pasting properties of fiber/wheat flour mixtures: initial viscosity (IV), pasting temperature (T_{past}), peak viscosity (V_{peak}), peak temperature (T_{peak}), holding strength (HS), final viscosity (final), breakdown (BD), setback from peak (SB_{peak}), total setback (SB_{total}) (n=3).

	Fiber conc. (g/100 g flour)	IV (mPa.s)	T_{past} (°C)	V_{peak} (mPa.s)	T_{peak} (°C)	HS (mPa.s)	V_{final} (mPa.s)	BD (mPa.s)	SB_{peak} (mPa.s)	SB_{total} (mPa.s)
PF	0	2.70 ± 0.04^{a}	56.3 ± 0.3^{a}	2705 ± 112^{ad}	92.20 ± 0.08^{a}	962 ± 36^{abc}	2420 ± 96^{ac}	1743 ± 76^{a}	-296 ± 6^{a}	1458 ± 61^{ad}
	1	2.96 ± 0.07^{ac}	57.4 ± 0.9^{ab}	2649 ± 23^{acd}	92.08 ± 0.03^{acd}	962 ± 28^{abc}	2381 ± 42^{ac}	1687 ± 22^{ac}	-267 ± 26^{ab}	1420 ± 18^{ad}
	5	4.10 ± 0.04^{abd}	58.0 ± 0.7^{abc}	2403 ± 74^{b}	92.03 ± 0.08^{ab}	911 ± 32^{a}	2179 ± 75^{b}	1493 ± 42^{b}	-224 ± 11^{abcde}	1269 ± 44^{b}
	10	5.90 ± 0.13^{bd}	57.8 ± 0.5^{ab}	2505 ± 29^{ab}	91.97 ± 0.06^{ab}	998 ± 14^{bc}	2299 ± 29^{ab}	1507 ± 31^{b}	-206 ± 30^{bcde}	1301 ± 17^{bc}
CF	0	2.70 ± 0.04^{a}	56.3 ± 0.3^{a}	2705 ± 112^{ad}	92.20 ± 0.08^{a}	962 ± 36^{abc}	2420 ± 96^{ac}	1743 ± 76^{a}	-296 ± 6^{a}	1458 ± 61^{ad}
	1	3.97 ± 0.74^{ab}	57.6 ± 0.9^{ab}	2578 ± 40^{abd}	92.07 ± 0.27^{ac}	936 ± 22^{ab}	2351 ± 23^{abc}	1643 ± 31^{ab}	-228 ± 29^{abcd}	1415 ± 1^{acd}
	5	4.54 ± 0.23^{bcd}	58.7 ± 0.1^{bc}	2464 ± 22^{bc}	92.19 ± 0.10^{ac}	942 ± 23^{ab}	2330 ± 39^{abd}	1523 ± 5^{bc}	-161 ± 35^{cde}	1388 ± 17^{abe}
	10	7.08 ± 0.16^{d}	59.1 ± 0.2^{c}	3350 ± 68^{d}	92.27 ± 0.09^{a}	1341 ± 33^{c}	3100 ± 64^{c}	2009 ± 39^{a}	-251 ± 17^{abc}	1759 ± 32^{de}
AF	0	2.70 ± 0.04^{a}	56.3 ± 0.3^{a}	2705 ± 112^{ad}	92.20 ± 0.08^{a}	962 ± 36^{abc}	2420 ± 96^{ac}	1743 ± 76^{a}	-296 ± 6^{a}	1458 ± 61^{ad}
	1	3.50 ± 0.28^{ac}	57.2 ± 0.5^{ab}	2565 ± 117^{abe}	91.60 ± 0.19^{b}	919 ± 16^{a}	2320 ± 81^{ab}	1646 ± 102^{ab}	-245 ± 45^{abcd}	1401 ± 65^{abd}
	5	4.07 ± 0.35^{ab}	58.3 ± 0.8^{bc}	2458 ± 46^{bc}	91.89 ± 0.02^{bc}	962 ± 18^{abc}	2297 ± 36^{ab}	1496 ± 29^{b}	-161 ± 17^{de}	1335 ± 18^{ab}
	10	5.27 ± 0.35^{bd}	57.9 ± 0.5^{bc}	2764 ± 65^{de}	91.73 ± 0.03^{bd}	1215 ± 21^{bc}	2739 ± 59^{cd}	1549 ± 48^{bc}	-26 ± 10^{de}	1523 ± 43^{de}

$^{a-e}$ Results marked by the same letter within the same column are not statistically different ($p > 0.05$).

foods

MDPI

Table S3. Impact of pea (PF), cocoa (CF), and apple (AF) fiber on bread properties: moisture, baking loss, density, and color parameters of the crust and crumb [L, a, and b] (n=3).

					Color Crust			Color Crumb		
	**Fiber conc. (g/100 g flour)**	**Moisture (%)**	**Baking loss (%)**	**Density (g/mL)**	**L**	**a**	**b**	**L**	**a**	**b**
PF	0	45.1 ± 0.4^{a}	14.3 ± 1.4^{ac}	0.40 ± 0.03^{ac}	64.3 ± 1.8^{a}	11.0 ± 1.5^{ac}	34.7 ± 1.9^{ab}	67.6 ± 1.4^{ab}	1.0 ± 0.0^{a}	17.3 ± 0.2^{ab}
	1	46.0 ± 1.2^{acd}	16.2 ± 1.6^{a}	0.38 ± 0.02^{ac}	59.1 ± 1.6^{ab}	14.8 ± 0.4^{b}	37.3 ± 0.1^{a}	69.0 ± 1.7^{ad}	1.1 ± 0.1^{a}	17.6 ± 0.4^{abc}
	5	47.8 ± 0.2^{b}	13.2 ± 0.7^{b}	0.50 ± 0.03^{b}	67.6 ± 5.8^{a}	8.7 ± 1.9^{a}	34.6 ± 1.8^{ab}	72.3 ± 1.0^{a}	1.4 ± 0.1^{a}	18.2 ± 0.3^{ac}
CF	0	45.1 ± 0.4^{a}	14.3 ± 1.4^{ac}	0.40 ± 0.03^{ac}	64.3 ± 1.8^{a}	11.0 ± 1.5^{ac}	34.7 ± 1.9^{ab}	67.6 ± 1.4^{ab}	1.0 ± 0.0^{a}	17.3 ± 0.2^{ab}
	1	45.8 ± 0.8^{ac}	12.9 ± 1.4^{ab}	0.37 ± 0.02^{a}	53.5 ± 1.3^{bc}	12.1 ± 0.2^{ab}	29.9 ± 0.8^{bc}	52.6 ± 3.2^{bc}	6.0 ± 0.5^{bc}	17.3 ± 0.9^{ab}
	5	47.1 ± 0.1^{bc}	12.9 ± 1.4^{bc}	0.46 ± 0.03^{bc}	41.6 ± 0.5^{b}	12.7 ± 0.3^{bc}	20.8 ± 0.1^{c}	36.8 ± 0.9^{cd}	10.2 ± 0.3^{b}	16.5 ± 0.8^{b}
AF	0	45.1 ± 0.4^{a}	14.3 ± 1.4^{ac}	0.40 ± 0.03^{ac}	64.3 ± 1.8^{a}	11.0 ± 1.5^{ac}	34.7 ± 1.9^{ab}	67.6 ± 1.4^{ab}	1.0 ± 0.0^{a}	17.3 ± 0.2^{ab}
	1	45.9 ± 0.2^{acd}	14.3 ± 0.0^{ab}	0.40 ± 0.01^{ac}	61.5 ± 0.5^{ac}	10.9 ± 1.3^{ac}	34.6 ± 2.2^{ab}	62.7 ± 1.2^{ac}	1.9 ± 0.1^{a}	16.7 ± 0.2^{b}
	5	47.2 ± 1.0^{bd}	12.9 ± 1.4^{b}	0.48 ± 0.01^{b}	55.6 ± 0.8^{bc}	11.4 ± 0.9^{ac}	32.2 ± 1.0^{bc}	54.2 ± 1.4^{bcd}	4.4 ± 0.1^{ac}	19.3 ± 0.3^{c}

[a-d] Results marked by the same letter within the same column are not statistically different ($p > 0.05$).

foods MDPI

Table S4. Impact of pea (PF), cocoa (CF), and apple (AF) fiber on Texture Profile Analysis (TPA) parameters [hardness, cohesiveness, chewiness, springiness, and resilience] (n=3).

	Fiber conc. (g/100 g flour)	TPA Hardness (g)	Springiness (-)	Cohesiveness (-)	Chewiness (g)	Resilience (-)
PF	0	1231 ± 137[ac]	0.91 ± 0.02[a]	0.74 ± 0.01[a]	819 ± 79[ac]	0.40 ± 0.01[a]
	1	1263 ± 322[ab]	0.92 ± 0.04[a]	0.77 ± 0.02[ab]	868 ± 192[ab]	0.44 ± 0.03[ab]
	5	1850 ± 142[b]	0.88 ± 0.02[b]	0.74 ± 0.01[a]	1206 ± 78[b]	0.41 ± 0.01[a]
CF	0	1231 ± 137[ac]	0.91 ± 0.02[a]	0.74 ± 0.01[a]	819 ± 79[ac]	0.40 ± 0.01[a]
	1	1072 ± 79[ac]	0.91 ± 0.14[ab]	0.78 ± 0.00[b]	768 ± 58[ac]	0.47 ± 0.01[b]
	5	1177 ± 132[ac]	0.90 ± 0.01[ab]	0.77 ± 0.01[ab]	811 ± 72[ac]	0.45 ± 0.01[ab]
AF	0	1231 ± 137[ac]	0.91 ± 0.02[a]	0.74 ± 0.01[a]	819 ± 79[ac]	0.40 ± 0.01[a]
	1	923 ± 112[c]	0.90 ± 0.02[ab]	0.80 ± 0.02[b]	662 ± 61[c]	0.46 ± 0.02[b]
	5	1389 ± 247[ab]	0.90 ± 0.03[ab]	0.79 ± 0.01[b]	978 ± 115[ab]	0.47 ± 0.01[b]

[a-c] Results marked by the same letter within the same column are not statistically different ($p > 0.05$).
